# Retinocochleocerebral Vasculopathy Presented as Bilateral Neovascular Glaucoma

**DOI:** 10.1002/ccr3.70051

**Published:** 2025-01-02

**Authors:** Mehrdad Motamed Shariati, Farid Shekarchian, Mariye Yaghoubi

**Affiliations:** ^1^ Eye Research Center Mashhad University of Medical Sciences Mashhad Iran

**Keywords:** branched retinal artery occlusion, Cochlear infarction, Neovascular glaucoma, Susac syndrome

## Abstract

Susac is a rare systemic disease characterized by ischemic events involving the cochlea, brain, and retina. Delay in the diagnosis leads to sight‐threatening complications such as neovascular glaucoma.

## 1 Introduction

Retinocochleocerebral vasculopathy, a rare microangiopathic disease affecting the retina, cochlea, and brain, is also known as Susac syndrome. The clinical trio of encephalopathy, branch retinal artery occlusion (BRAO), and sensorineural hearing loss was first reported by John O. Susac in 1979 [1, 2, 3, 4]. The syndrome mostly affects young women, usually in their third or fourth decade of life, although cases in both sexes and a wider age range have been reported [2, 5]. Susac syndrome can be challenging to diagnose due to its rarity and variable presentation, leading to delays in diagnosis and treatment.

Although the exact cause of Susac syndrome is unknown, it is thought to be related to an immune‐mediated endotheliopathy that affects the microvasculature. The typical symptoms arise from the obstruction of the small arterioles in the brain, retina, and cochlea. The extent and site of vascular involvement result in neurological, ocular, and auditory symptoms. Retinal artery occlusions are usually the cause of visual impairment along with neurological symptoms, such as headache, disorientation, memory loss, and even localized neurological deficits. Unilateral or bilateral sudden hearing loss is a common symptom of auditory involvement [3].

This case report describes a distinctive presentation of Susac syndrome, in which bilateral neovascular glaucoma was the first indication of the condition.

## 2 Case History and Examination

**FIGURE 1:**
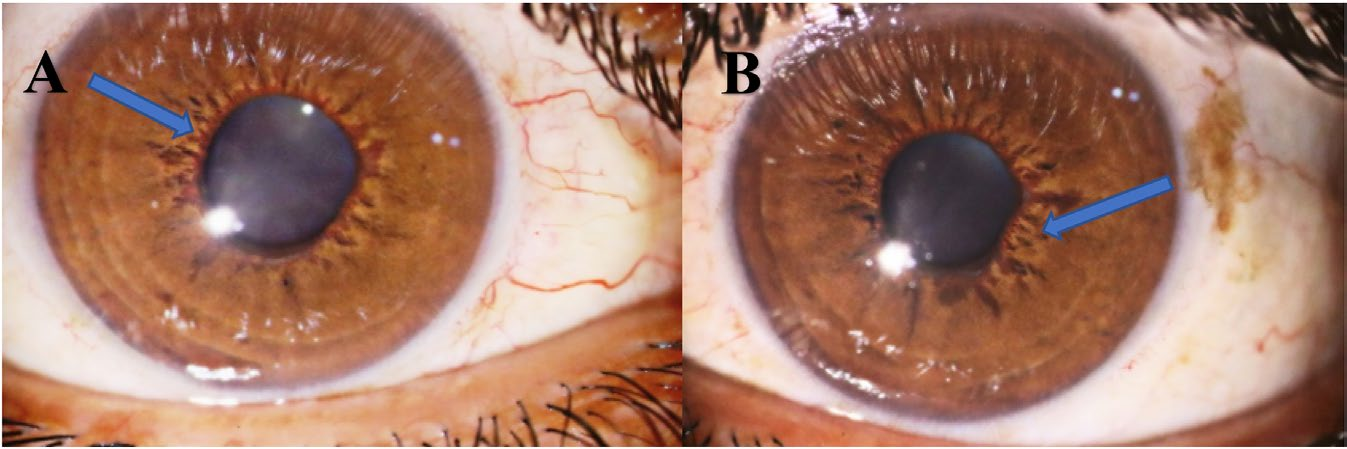
The slit photograph of the patient's eyes shows neovascularization of the iris in the right (A) and the left eye (B) (blue arrows).

**FIGURE 2:**
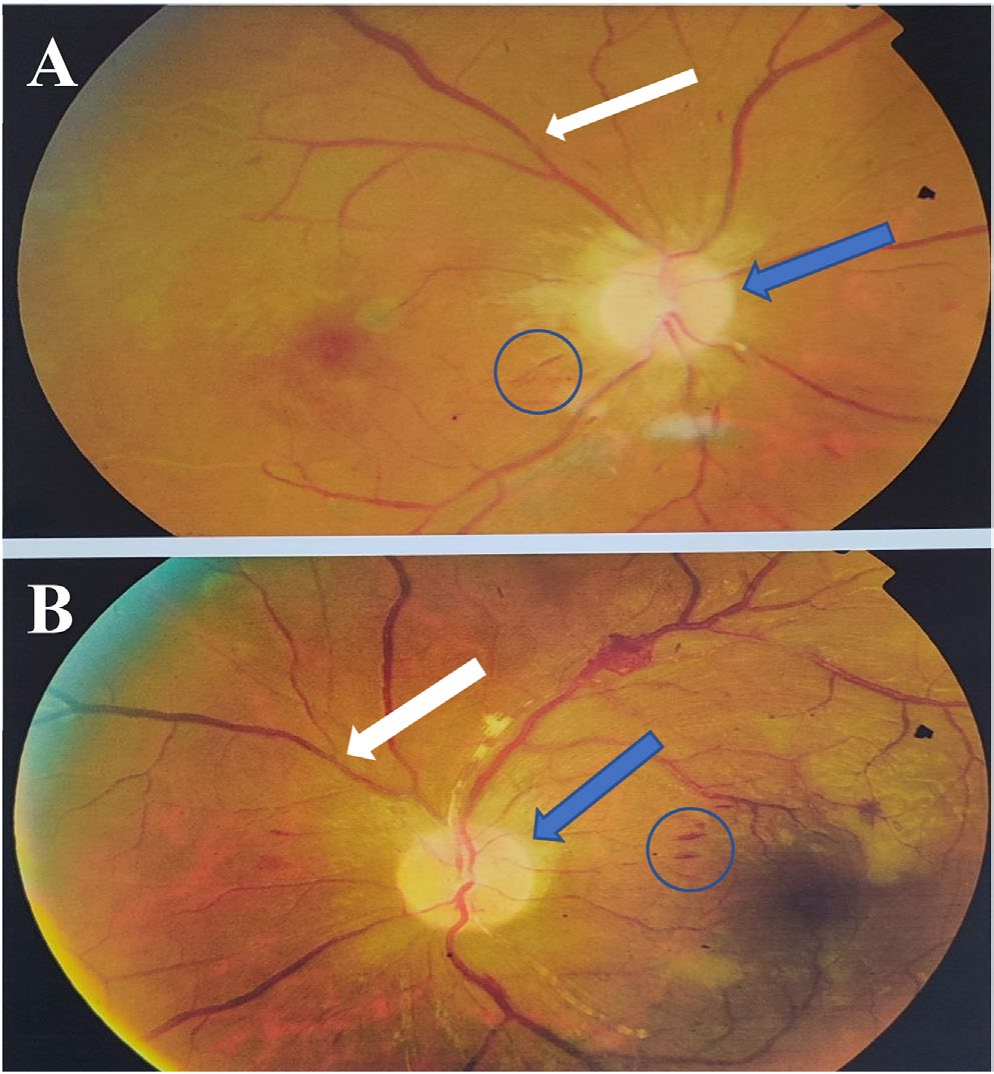
Fundus photographs showed optic disc paleness (blue arrows), splinter hemorrhages (circles), and vascular focal narrowing and occlusions (white arrows) in the right (A) and the left eye (B).

A 34‐year‐old man presented with acute painful visual loss in both eyes accompanied by tinnitus and hearing loss from 4 weeks ago. He had no history of diabetes mellitus, hypertension, or cardiac disease. The best‐corrected distance visual acuity (BCDVA) was counting fingers (CF) at 1.5 m for the right eye (RE) and 6/10 for the left eye (LE). The relative afferent pupillary defect was positive for the right eye. The intraocular pressure (IOP) was 30 and 25 mmHg for the RE and LE, respectively. The anterior segment examination showed neovascularization of the iris (NVI) in both eyes (Figure 1). Funduscopic examination revealed bilateral optic disc and retinal paleness, splinter hemorrhages, and vascular focal narrowing and occlusions (Figure 2).

## 3 Methods

**FIGURE 3:**
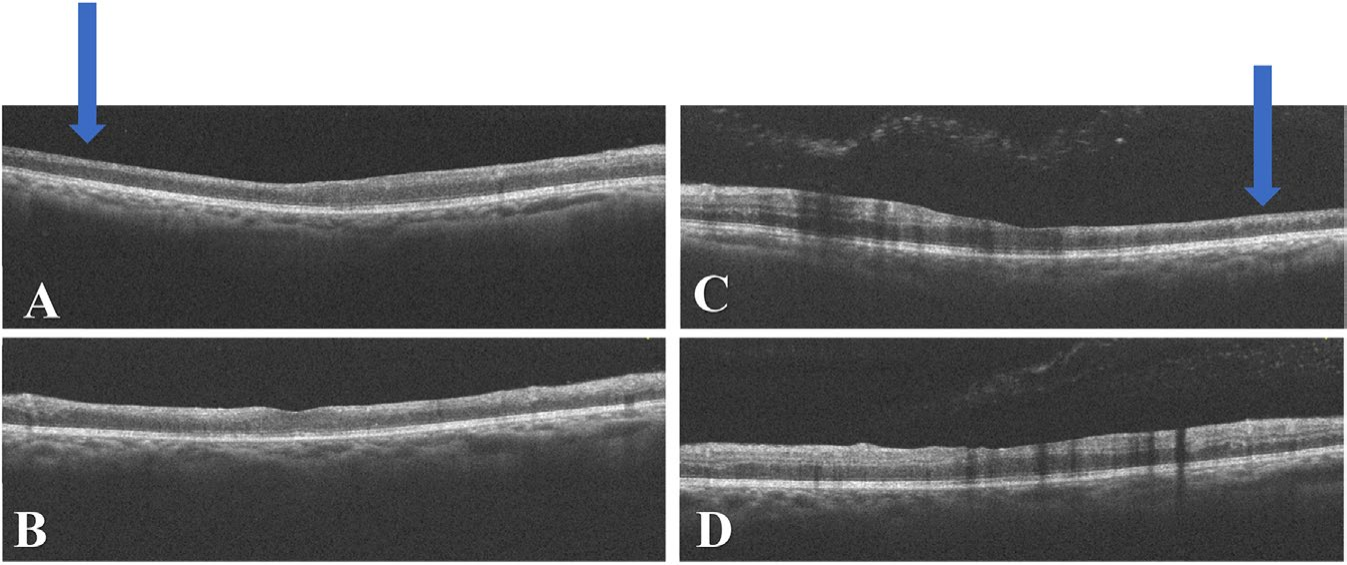
SD‐OCT image of the right (A, B) and the left eye (C, D) revealed inner retinal hyperreflectivity and atrophy of the neurosensory retina (blue arrows).

**FIGURE 4:**
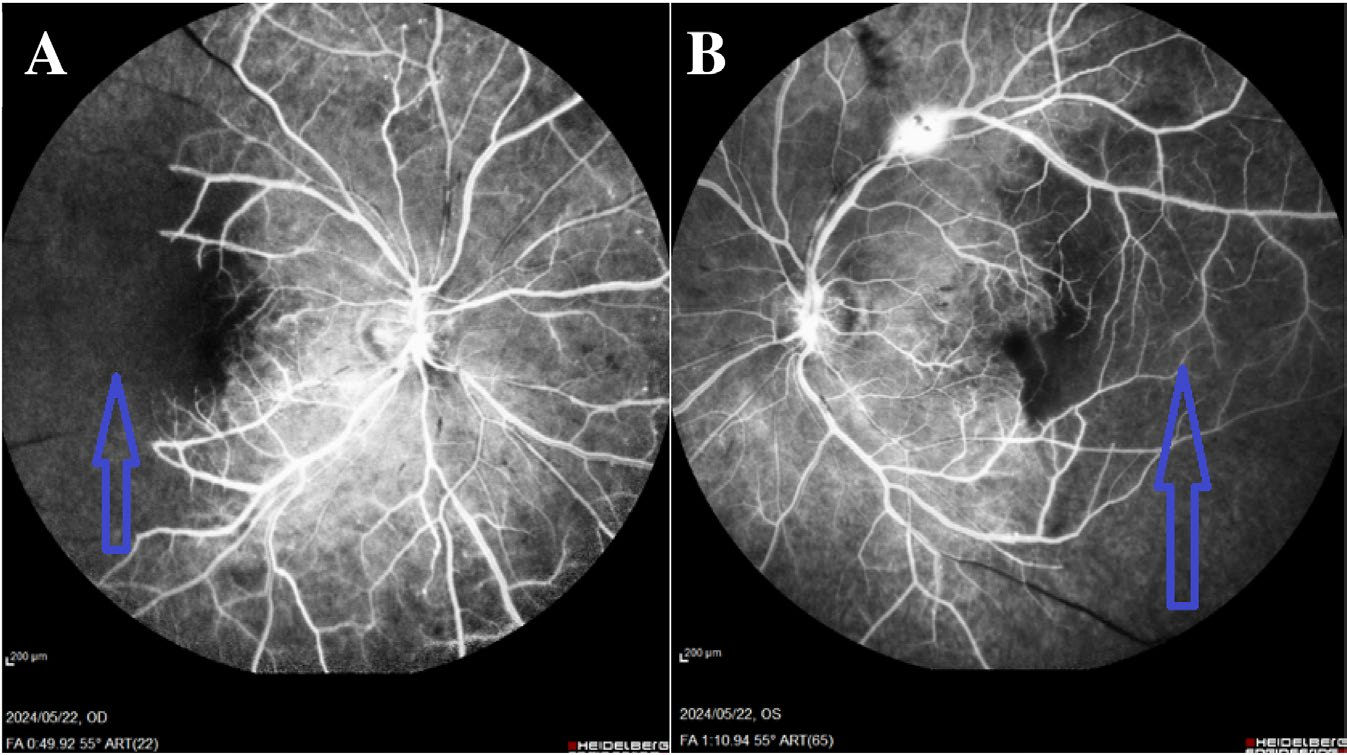
Fundus fluorescein angiography shows large areas of capillary nonperfusion (blue arrows) and perivascular staining in the right (A) and the left eye (B).

**FIGURE 5:**
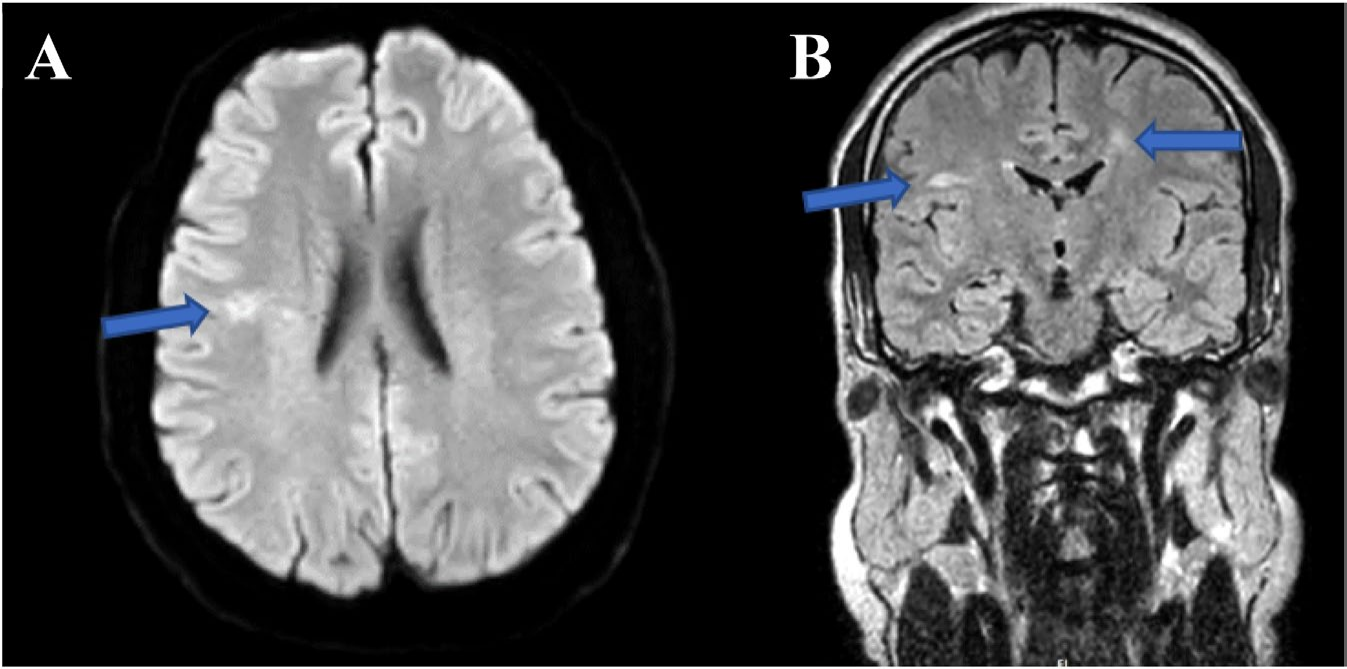
MRI T2 weighted axial (A) and coronal (B) images revealed hyperintense white matter lesions (Blue arrows).

We used multimodal imaging to evaluate the patient further. Spectral‐domain optical coherence tomography (SD‐OCT) (Heidelberg Eye Explorer version 1.9.13.0, Spectralis Viewing Module 6.5.2.0; Heidelberg Engineering) demonstrated inner retinal hyperreflectivity and atrophy of the neurosensory retina in both eyes (Figure 3). The results of fundus fluorescein angiography (FAG) (Heidelberg Eye Explorer version 1.9.13.0, Spectralis Viewing Module 6.5.2.0; Heidelberg Engineering) showed large areas of capillary nonperfusion and perivascular staining (Figure 4). Our evaluations suggested bilateral neovascular glaucoma and multiple retinal artery branch occlusions. Regarding these findings, the Susac syndrome was considered at the top of the differential diagnosis list. A neurology consultation was performed. Based on previous symptoms and headaches; the neurologist underwent an MRI with contrast on our patient. The contrast‐enhanced MRI showed numerous enhancing bilateral white matter microinfarcts (Figure 5). Although no additional neurological deficits were observed on clinical examination, MRI findings revealed numerous bilateral white matter microinfarcts. This imaging finding supports a diagnosis of Susac syndrome, as it aligns with the characteristic small‐vessel vasculopathy affecting the central nervous system. In this case, the absence of overt neurological symptoms highlights the variable presentation of Susac syndrome, which can initially manifest predominantly with ocular and auditory symptoms, underscoring the importance of imaging in early diagnosis.

## 4 Conclusion and Results

**FIGURE 6:**
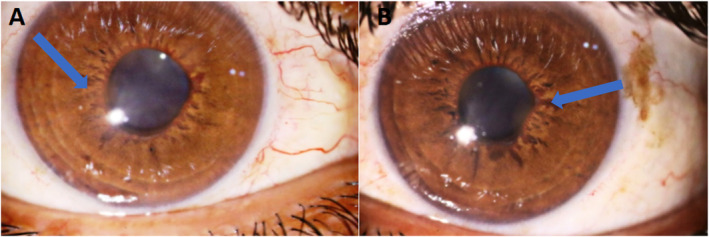
The slit photograph shows partially regressed iris neovascularization of the right (A) and the left eye (B) after treatment initiation.

Neovascular glaucoma was treated with topical antiglaucoma medications (Eye drop brimonidine 0.2%, timolol 0.5% every 12 h, and tablet acetazolamide at 250 mg every 8 h), intravitreal bevacizumab injection (1.25 mg/0.05 mL), and targeted retinal photocoagulation. Oral prednisolone was started with a dose of 50 mg/day. At the last follow‐up visit, three weeks after the first presentation, the patient's visual acuity was improved to 2/10 and 8/10 for the RE and the LE, respectively. The NVIs were partially regressed (Figure 6). A rheumatology consultation was requested to start immunomodulation therapy (IMT). A rheumatology consultation was requested to initiate immunomodulation therapy (IMT) due to the complex selection and monitoring of these treatments in patients with immune‐mediated conditions like Susac syndrome. Rheumatologists provide specialized expertise in tailoring therapy based on the disease severity, systemic involvement, and patient‐specific factors, ensuring optimal efficacy while minimizing potential adverse effects. This multidisciplinary approach is essential for comprehensive management, particularly in suspected or confirmed systemic immune involvement cases.

## 5 Discussion

A complicated and uncommon symptom of multisystem vascular disease, the case of retinocochleocerebral vasculopathy presenting as bilateral neovascular glaucoma (NVG) highlights the intricate relationship between systemic vascular pathology and localized ocular manifestations. This case underscores the significance of a multidisciplinary approach to diagnosis and treatment as it encompasses several organ systems and carries a high risk of severe morbidity.

Retinocochleocerebral vasculopathy is a rare genetic disorder characterized by systemic small‐vessel vasculopathy affecting the retina, cochlea, and brain. The TREX1 gene mutations have been reported [6]. TREX1 plays a critical role in DNA repair. Retinal vasculopathy, sensorineural hearing loss, and a variety of cerebral symptoms, including leukoencephalopathy, stroke, and cognitive decline, are the disease's prominent characteristics. Immune‐mediated endothelial damage is a pathogenesis that results in microvascular occlusions and ischemia in the affected organs [3, 5].

Neovascular glaucoma (NVG) is a devastating form of secondary glaucoma characterized by the formation of abnormal neovessels on the iris and the anterior chamber angle, leading to increased intraocular pressure (IOP) and progressive optic neuropathy. For NVG to occur in the setting of retinocochleocerebral vasculopathy, persistent retinal ischemia is usually the cause. Ischemia and hypoxia brought on by retinal vasculopathy trigger the production of proangiogenic factors, especially vascular endothelial growth factor (VEGF). The overexpression of VEGF induces neovascularization in the anterior portion of the eye and the retina, resulting in NVG [7, 8].

The bilateral appearance of NVG in this particular case emphasizes how severe and broad the underlying vasculopathy is. The involvement of both eyes suggests a systemic process rather than a localized ocular disease.

A young patient presenting with bilateral NVG should raise suspicion of underlying systemic vasculopathy and other medical conditions that may worsen retinal ischemia. Eales' disease, sickle cell retinopathy, diabetic retinopathy, central retinal vein occlusion (CRVO), and ocular ischemic syndrome are the primary differential diagnoses [8]. In this case, systemic symptoms like headache and tinnitus could indicate susac syndrome among other potential differential diagnoses.

The aggressive course of NVG combined with the progressive nature of the underlying systemic disease makes managing NVG in the setting of retinocochleocerebral vasculopathy challenging. The main objectives of treatment include addressing the underlying ischemic process, managing neovascularization, and controlling IOP.

Aqueous suppressants such as beta‐blockers, carbonic anhydrase inhibitors, and alpha agonists are commonly used in medical treatment to decrease IOP. However, these drugs often only provide temporary relief and may not be adequate for long‐term disease management [8].

The use of anti‐VEGF agents, such as bevacizumab, has shown promise in managing neovascularization by inhibiting the angiogenic stimulus. Intravitreal injections of anti‐VEGF agents can reduce the extent of neovascularization and decrease IOP. However, the effects are often temporary, and repeated injections are necessary. Another key component of NVG treatment is pan‐retinal photocoagulation (PRP), which targets the ischemic retina and lowers VEGF production. Advanced cases requiring surgical treatments such as trabeculectomy or glaucoma drainage devices may be necessary when medication and laser therapy are unable to control IOP. However, because of the inflammatory nature of the disease and the existence of neovascularization, these procedures are considered high risk regarding failure and complications [8].

Given the systemic nature of retinocochleocerebral vasculopathy, collaboration with other specialties, such as neurology and rheumatology, is essential. Systemic immunosuppressive therapy may be considered to address the underlying vasculitis and reduce the progression of systemic and ocular involvement. Corticosteroids, cytotoxic agents, or biologic therapies targeting specific inflammatory pathways may be utilized, although the evidence for their efficacy in retinocochleocerebral vasculopathy is limited [9, 10].

The prognosis for patients with retinocochleocerebral vasculopathy presenting with NVG is generally poor, especially when the condition is advanced at the time of diagnosis. The progressive nature of the vasculopathy, combined with the aggressive course of NVG, often leads to significant visual impairment or blindness despite treatment. Early diagnosis and intervention are crucial in preserving vision and preventing further systemic complications [10].

This case underscores the significance of evaluating systemic vasculopathy in individuals with bilateral NVG, particularly when systemic symptoms are present. Collaborative management and a personalized strategy addressing both ocular and systemic manifestations are crucial for enhancing outcomes in such cases.

## Author Contributions

All authors contributed significantly to this report and agree to be accountable for all aspects of the work. All authors read and approved the final manuscript.

## Ethics Statement

The authors have nothing to report.

## Consent

Written informed consent was obtained from the patient to publish this case report and any accompanying images. A copy of the written consent is available for review by the Editor‐in‐Chief of this journal.

## Conflicts of Interest

The authors declare no conflicts of interest.

## Data Availability

The datasets used during the current study are available from the corresponding author upon reasonable request.
